# An Observational Pilot Study Evaluating the Utility of Minimally Invasive Tissue Sampling to Determine the Cause of Stillbirths in South African Women

**DOI:** 10.1093/cid/ciz573

**Published:** 2019-10-09

**Authors:** Shabir A Madhi, Jayani Pathirana, Vicky Baillie, Clare Cutland, Yasmin Adam, Alane Izu, Quique Bassat, Dianna M Blau, Robert F Breiman, Martin Hale, Siobhan Johnstone, Roosecelis B Martines, Azwifarwi Mathunjwa, Susan Nzenze, Jaume Ordi, Pratima L Raghunathan, Jana M Ritter, Fatima Solomon, Jeannette Wadula, Sherif R Zaki, Richard Chawana

**Affiliations:** 1 Medical Research Council: Respiratory and Meningeal Pathogens Research Unit, University of the Witwatersrand, Faculty of Health Science, Johannesburg, South Africa; 2 Department of Science and Technology/National Research Foundation: Vaccine Preventable Diseases, University of the Witwatersrand, Faculty of Health Sciences, Johannesburg, South Africa; 3 Department of Obstetrics and Gynaecology, Chris Hani Baragwanath Academic Hospital, School of Clinical Medicine, Faculty of Health Sciences, University of the Witwatersrand, Johannesburg, South Africa; 4 ISGlobal, Hospital Clínic, Universitat de Barcelona, Barcelona, Spain; 5 Centro de Investigação em Saúde de Manhiça (CISM), Maputo, Mozambique; 6 Catalan Institution for Research and Advanced Studies (ICREA), Barcelona, Spain; 7 Pediatric Infectious Diseases Unit, Pediatrics Department, Hospital de Sant Joan de Deu, University of Barcelona, Barcelona, Spain; 8 Consorcio de Investigacion Biomedica en Red de Epidemiologia y Salud, Madrid, Spain; 9 Center for Global Health, Centers for Disease Control and Prevention, Atlanta, Georgia, USA; 10 Emory Global Health Institute, Emory University, Atlanta, Georgia, USA; 11 National Health Laboratory Service, Department of Anatomical Pathology, School of Pathology, University of the Witwatersrand, Faculty of Health Sciences, Johannesburg, South Africa; 12 Infectious Diseases Pathology Branch, Division of High-Consequence Pathogens and Pathology, National Center for Emerging and Zoonotic Infectious Diseases, Centers for Disease Control and Prevention, Atlanta, Georgia, USA; 13 National Health Laboratory Service, Department of Microbiology and Infectious Diseases, School of Pathology, Faculty of Health Sciences, University of the Witwatersrand, Johannesburg, South Africa

**Keywords:** MITS, stillbirth and fetal death, maternal hypertension, fetal hypoxia, fetal infection

## Abstract

**Background:**

Despite approximately 2.6 million stillbirths occurring annually, there is a paucity of systematic biological investigation and consequently knowledge on the causes of these deaths in low- and middle-income countries (LMICs). We investigated the utility of minimally invasive tissue sampling (MITS), placental examination, and clinical history, in attributing the causes of stillbirth in a South African LMIC setting.

**Methods:**

This prospective, observational pilot study undertook sampling of brain, lung, and liver tissue using core biopsy needles, blood and cerebrospinal fluid collection, and placental examination. Testing included microbial culture and/or molecular testing and tissue histological examination. The cause of death was determined for each case by an international panel of medical specialists and categorized using the World Health Organization’s *International Classification of Diseases, Tenth Revision* application to perinatal deaths.

**Results:**

A cause of stillbirth was identifiable for 117 of 129 (90.7%) stillbirths, including an underlying maternal cause in 63.4% (n = 83) and an immediate fetal cause in 79.1% (n = 102) of cases. The leading underlying causes of stillbirth were maternal hypertensive disorders (16.3%), placental separation and hemorrhage (14.0%), and chorioamnionitis (10.9%). The leading immediate causes of fetal death were antepartum hypoxia (35.7%) and fetal infection (37.2%), including due to *Escherichia coli* (16.3%), *Enterococcus* species (3.9%), and group B *Streptococcus* (3.1%).

**Conclusions:**

In this pilot, proof-of-concept study, focused investigation of stillbirth provided granular detail on the causes thereof in an LMIC setting, including provisionally highlighting the largely underrecognized role of fetal sepsis as a dominant cause.

## Teaser

Minimally invasive tissue sampling, placental examination, and clinical history were utilized to attribute cause of death in South African stillbirths. The leading underlying maternal causes included hypertensive disorders, hemorrhage, and chorioamnionitis, and immediate fetal causes were antepartum hypoxia and infection.

In 2015, the estimated number of stillbirths (2.6 million) approximated neonatal deaths (2.7 million) [1], 98% of which occurred in low- and middle-income countries (LMICs). Furthermore, the annual rate reduction of stillbirths from 2000 to 2015 was 2%, which was lower than rate reduction in neonatal mortality (3.1%) and deaths in children aged 1–59 months (4.7%) [2, 3]. The United Nations Children’s Fund–adopted “Every Newborn Action Plan” aspires to reduce stillbirth rates from 18.4 per 1000 births in 2015 to 12 per 1000 births by 2030 [4]. The paucity of systematic investigation and consequent limited understanding of the causes of stillbirth in LMICs is recognized as a major impediment in achieving this goal.

Currently, estimates on causes of stillbirth in LMICs are premised on limited vital registration and verbal autopsy data, coupled with modeling of known risk-factor prevalence [1]. The ubiquitous absence of biological investigation of stillbirths in LMICs is multifactorial—resources are limited and a significant proportion of cases occur at home and thus outside of the health system—and is compounded by cultural issues related to acceptability of postmortem autopsies [5]. Detailed investigation on the causes of stillbirths was undertaken by the Stillbirth Collaborative Research Network (SCRN) in a multicenter study in the United States between 2006 and 2008, when the stillbirth rate was 6 per 1000 births. The most informative tools in attributing the cause of stillbirth in the SCRN study were maternal medical record review, placental examination and histology (52.3%), complete diagnostic autopsy (CDA, which included bacterial culture of tissue samples; 31.4%), and karyotyping (9%) [6].

To overcome the obstacles of undertaking CDA in LMICs, a Mozambican study compared the utility of minimally invasive autopsy (MIA) to CDA for attributing the cause of stillbirths and neonatal deaths [7]. A cause of stillbirth was attributed to 89% of 18 stillbirths investigated by CDA and in 83% based on MIA findings. There was moderate concordance (κ = 0.78 [95% confidence interval, .56–.99]) in the cause of death (CoD) attribution between the 2 methods, despite the absence of placental examination and not using any clinical information when evaluating for the CoD.

The aim of our study was to pilot the utility of minimally invasive tissue sampling (MITS, also referred to as MIA), together with maternal medical record review and placental macroscopic and histological examination, in attributing the cause of stillbirths in an LMIC setting in South Africa.

## METHODS

### Study Population and Site

The study population and study methods are described in the companion article on neonatal deaths in this supplement [8]. In brief, the study was done at Chris Hani Baragwanath Academic Hospital (CHBAH), Soweto, a secondary-tertiary hospital, where approximately 75% of 28 000 annual births in Soweto occur. CHBAH was the only public hospital at the time of the study in Soweto (unpublished administrative data, Obstetrics Department, CHBAH). While additional deliveries occur at 5 midwife-operated units in Soweto, women who are deemed to be high risk according to national guidelines are referred to a hospital, including women in preterm labor, those presenting with a stillbirth, or who had a previous stillbirth. The stillbirth rate in Soweto was 22.5 per 1000 births in 2016, which is higher than that estimated for South Africa based on vital registration data (14 per 1000 births in 2015) [2].

### Study Design

This prospective, observational study enrolled stillbirths delivered at CHBAH from 20 July 2015 to 8 August 2016. Stillbirths were defined as absence of any signs of life diagnosed either before delivery by sonography, or at birth by the attending clinician/midwife. In this study, we limited enrollment to stillbirths weighing ≥1000 g based on World Health Organization (WHO) reporting criteria [4]. Furthermore, we prioritized stillbirths from women who had consented to partake in a parallel observational study assessing for serocorrelates of protection against invasive group B *Streptococcus* (GBS) disease (ClinicalTrials.gov registration number: NCT02215226), followed by women who consented to study participation after confirmation of an intrauterine death or delivery of a stillborn baby. Assessment of gestational age of the stillbirths was based on the last menstrual period or on ultrasound findings performed as standard of care.

The study staff retrospectively abstracted clinical information from maternal medical records, including details on the mother, antenatal care, and labor history using a standard template. Stillbirths were categorized by timing of fetal death: antepartum, that is, fetal death confirmed by ultrasonography prior to the onset of labor; or intrapartum if there was evidence of fetal life during active labor by physical examination, cardiotocography, or ultrasonography.

### Minimally Invasive Tissue Sampling

Trained study staff undertook the MITS procedure. Detail of the methods used for undertaking MITS, including histological evaluation, is described elsewhere in this supplement [8]. In brief, this included multiple core tissue biopsies of the brain, left and right lung, and liver. The first tissue sample from each site was sent for culture (lung and liver), the second sample was used for molecular microbiological tests (lung only), and 6 subsequent tissue samples were sent for histopathology (brain, lung, and liver). Microbial culture and molecular testing were done on blood and cerebrospinal fluid (CSF) samples.

### Placental Examination

Following delivery, the placenta was retrieved and weighed prior to resecting a wedge of the parenchymal tissue and chorioamniotic membranes, which was placed in a sterile container for bacterial microscopy and culture. The rest of the placenta was then immersed in 10% buffered formalin and transported to Lancet Laboratories (Johannesburg, South Africa). Placental examination included macroscopic visualization, following which the histopathologist selected portions of placenta, which were embedded in paraffin and processed for routine hematoxylin and eosin staining using standard protocols [9].

### Molecular Tests

Details of the molecular testing for organisms presence in blood, lung, and CSF samples is as described in the companion manuscript on neonatal deaths [8]. The testing used a commercially available multiplex polymerase chain reaction (PCR) assays (Fast-Track Diagnostics [FTD], Sliema, Malta), and was done at the Respiratory and Meningeal Pathogens Research Unit laboratory. CSF samples were tested using the FTD-Neuro-9 meningitis panel, which included probes for cytomegalovirus (CMV), Epstein-Barr virus, adenovirus, herpes simplex virus 1 and 2, varicella zoster virus, enterovirus, parechovirus, human herpesvirus 6 and 7, and parvovirus B19. Lung and blood sample molecular testing was done using the FTD-Neonatal Sepsis kit, which included probes for CMV, group B *Streptococcus*, *Listeria monocytogenes*, *Escherichia coli*, *Staphylococcus aureus*, *Chlamydia trachomatis*, and *Ureaplasma urealyticum/parvum*. The molecular assays were not specifically designed for investigating infectious causes of stillbirths.

### Determination of Cause of Death

The CoD attribution was based on consensus opinion of a multidisciplinary international panel of specialists (listed under the Determination of Cause of Death [DeCoDe] panel, see Acknowledgments), as described in the Supplementary Materials and elsewhere [8]. The framework for reporting on the CoD was based on the 2016 WHO perinatal CoD reporting recommendations [10], which includes recording of the “underlying” condition which led to the sequence of events culminating in fetal demise, and the most “immediate” medical event that caused the fetal death.

### Statistical Analysis

Demographic and clinical data are summarized using means and standard deviations or medians and range where appropriate. Student *t* tests were performed to compare means and the Wilcoxon-rank sum test was used for nonnormal quantitative data. Fisher exact test was used for comparing categorical variables. Standardized weight scores were calculated using the International Fetal and Newborn Growth Consortium for the 21st Century standards and small for gestational age was defined as a standardized score (*z* score) < −2 [11].

### Ethical Considerations

The study was approved by the Human Research Ethics Committee (HREC 15021) of the University of the Witwatersrand, and registered at the South African National Department of Health Trials Registry (application number 3988). Grief counseling was administered to bereaved parents and guardians, following which they were informed of the study and provided an opportunity to discuss with other family members prior to consenting. The study staff continued providing grief counseling following the MITS, including when giving feedback on the cause of death.

## RESULTS

Overall, 129 stillbirths were enrolled, including 96 (74.4%) antepartum and 27 (20.9%) intrapartum cases; the timing of death was uncertain for 6 cases (Table 1). Seventy-seven percent of the women with stillbirths were referred to CHBAH from a surrounding midwife-obstetric unit. The median age of the women was 28 years, 17.6% were >35 years of age, and 96.1% were black Africans. The prevalence of maternal human immunodeficiency virus (HIV) and syphilis infection was 32.0% and 5.8%, respectively. Maternal pregnancy and labor-related complications included 23.1% with pregnancy-induced hypertension and 21.4% with antepartum hemorrhage. The median time between presentation to the hospital and delivery was 7.5 and 11.8 hours for antepartum and intrapartum stillbirths, respectively (Table 1).

**Table 1.** Maternal Demographic and Clinical Features of Antepartum and Intrapartum Stillbirths

Maternal Characteristics	Total (N = 129)^a^	Antepartum (n = 96)	Intrapartum (n = 27)	*P* Value^b^
Median age, y (range)	28 (17–42)	28 (17–42)	27.5 (20–42)	.73
Age >35 y	22/125 (17.6)	14/94 (14.9)	5/26 (19.2)	.56
Race				
Black African	124 (96.1)	93 (96.9)	26 (96.3)	
Other	5 (3.9)	3 (3.1)	1 (3.7)	> .99
HIV ELISA positive	41/128 (32.0)	31/95 (32.6)	10 (37.0)	.65
Syphilis positive on RPR	6/104 (5.8)	5/81 (6.2)	1/19 (5.3)	> .99
Gestation age at initial antenatal visit, wk (SD)	20.67 (6.53)	20.61 (6.75)	21 (5.6)	> .99
Pregnancy and labor complications^c^	n = 117	n = 90	n = 26	
Pregnancy-induced hypertension	27 (23.1)	19 (21.1)	8 (30.8)	.31
Antepartum hemorrhage	25 (21.4)	11 (12.2)	14 (53.8)	< .001
Placenta abruptio	20 (17.1)	9 (10.0)	11 (42.3)	< .001
Uterus rupture	3 (2.6)	1 (1.1)	2 (7.7)	.13
Twin pregnancy	7 (6.0)	4 (4.4)	3 (11.5)	.19
Received antibiotics within week of delivery	14/101 (13.9)	10/77 (13)	3/23 (13)	> .99
Referral from midwife-operated unit	85/111 (76.6)	64/86 (74.4)	21/25 (84)	.43
Mode of delivery				
Vaginal	91/124 (73.4)	72/94 (76.6)	17/26 (65.4)	
Emergency cesarean delivery	27/124 (21.8)	18/94 (19.1)	9/26 (34.6)	
Elective cesarean delivery	3/124 (2.4)	3/94 (3.2)	0/26 (0)	
Unspecified indication for cesarean delivery	3/124 (2.4)	1/94 (1.1)	0/26 (0)	.44
Median time between labor onset and delivery, h (range)	9.1 (0.8–76.7)	7.5 (0.8–76.7)	11.8 (0.8–29.6)	> .99
Placenta collected	99 (76.7)	77 (80.2)	21 (77.8)	.79

Data are presented as No. (%) unless otherwise indicated.

Abbreviations: ELISA, enzyme-linked immunosorbent assay; HIV, human immunodeficiency virus; RPR, rapid plasma reagin; SD, standard deviation.

^a^Included are 6 subjects without sufficient documentation to attribute whether antepartum or intrapartum death.

^b^Comparison of antepartum to intrapartum cases.

^c^Based on retrospective review of available maternal records with adequate documentation of information.

Two-thirds of the stillbirths had a macerated appearance, which expectedly was more common in antepartum (82.1%) than intrapartum (20.0%) cases (*P* < .001; Table 2). Gross congenital abnormalities were present in 3 stillbirths, including 1 case each of hydrocephalus and anencephaly, and the third being unspecified. The median birth weight and gestational age were 2070 g and 35 weeks, respectively, with 78.1% of stillbirths weighing >1500 g. Small for gestational age (SGA) was present in 17.6% of 102 cases in whom gestational age and birth weight data were available. Two of 41 (4.8%) stillbirths born to HIV-infected mothers had a reactive HIV type 1 PCR test at birth. Testing for HIV by PCR failed in 5 cases of antepartum stillbirths. Overall, the median time between delivery and MITS was 16.8 hours (Table 2).

**Table 2.** Demographic and Clinical Characteristics of Antepartum and Intrapartum Stillbirth Investigated by Minimally Invasive Tissue Sampling

Features	Total (N = 129)	Antepartum (n = 96)	Intrapartum (n = 27)	*P* Value^a^
Male sex	64/123^b^ (52.0)^c^	48/91 (52.7)	11/26 (42.3)	.38
Physical appearance^d^				
Fresh	35/109 (32.1)	15/84 (17.9)	20/25 (80.0)	
Macerated	74/109 (67.9)	69/84 (82.1)	5/25 (20.0)	< .001
Gross congenital abnormality	3/129 (2.3)	1/96 (1.0)	2/27 (7.4)	.12
Birth weight^e^				
Median, g (range)	2070 (1000–4025)	1950 (1000–3690)	2280 (1100–4025)	.68
1000–1499 g	28/128 (21.9)	22/95 (23.2)	6/27 (22.2)	
1500–2499 g	62/128 (48.4)	50/95 (52.6)	10/27 (37)	
≥2500 g	38/128 (29.7)	23/95 (24.2)	11/27 (40.7)	.09
Gestational age, wk^f^				
Median (range)	35 (22–42)	35 (23–42)	35 (22–42)	.83
22–27	9/110 (8.2)	4/81 (4.9)	4/23 (17.4)	
28–36	67/110 (60.9)	53/81 (65.4)	10/23 (43.5)	
37–41	32/110 (29.1)	23/81 (28.4)	8/23 (34.8)	
≥42	2/110 (1.8)	1/81 (1.2)	1/23 (4.3)	.07
Small for gestational age: *z* score < −2^g^	18/102 (17.6)	16/75 (21.3)	2/21 (9.5)	.22
Median time between delivery and MITS, h (range)	16.8 (1.1–97.2)	16.2 (1.1–97.2)	23.9 (1.6–79.7)	> .99

Data are presented as No. (%) unless otherwise indicated.

Abbreviation: MITS, minimally invasive tissue sampling.

^a^Comparison of antepartum to intrapartum cases.

^b^Six subjects without sufficient documentation to attribute whether antepartum or intrapartum death.

^c^All values in parentheses are a percentage of total observations given in the denominator.

^d^Data available for 109 cases in total, including 84 antepartum and 25 intrapartum cases.

^e^Data available for 128 cases in total, including 95 antepartum and 27 intrapartum cases.

^f^Gestational age available for 110 overall, including 81 antepartum and 23 intrapartum cases.

^g^Data available for 102 subjects with paired gestational age and birth weight, including 75 antepartum and 21 intrapartum cases.

### Placental Pathology and Tissue Sample Histopathology

Placentas were available for investigation in 99 (76.7%) stillbirths, including 77 (80.2%) antepartum, cases 21 (70.8%) intrapartum cases and one case in whom timing of stillbirth could not be determined (Supplementary Table 1). Macroscopic evidence of placenta infarction was evident in 26.3% and retroplacental hematoma in 31.2% of cases. Histologically, placental infarction was identified in 50.0% of cases. Of those in which the extent of infarction was quantified (n = 38), 28.9% had ≥20% parenchymal involvement. Chorioamnionitis was histologically evident in 27.6% (n = 27) of placentas, and of those graded, 31.8% had grade I, 40.9% grade II, and 27.3% grade III inflammatory cellular infiltrates. Umbilical cord abnormalities included presence of cord thrombosis (4.2%), cord knots (4.2%), funisitis (3.1%), and chorionic vasculitis (2.2%) (Supplementary Table 1). There were no significant differences in placental findings between antepartum and intrapartum stillbirths.

There was a low yield of adequate samples (<2.5% for any organ) for histopathologic investigation of fetal tissue (Supplementary Table 2). The majority of tissue samples were either autolyzed (40.6%–64.8%), had a suboptimal number of core tissues for investigation (0.8%–43.8%), or no targeted tissue was collected at all (13.3%–55.5%).

### Underlying Causes of Stillbirth

An underlying maternal cause of stillbirth was attributed by the DeCoDe panel for 64.3% (n = 83) of cases; with the level of certainty graded mainly as “confident” (90%) (Table 3). The DeCoDe panel could not attribute an underlying maternal condition in 35.7% of cases, including 24 (18.6%) cases in which although there was a maternal medical condition that was considered to be a possible risk factor for stillbirths, the DeCoDe panel adjudicated the condition not to have caused the stillbirth. This included 10 women with underlying HIV infection and 3 with multiple pregnancies. In a further 17.1% (n = 22) of cases, there was no attributable maternal condition identified to have caused the stillbirth (Table 3).

**Table 3.** Maternal Underlying Conditions and Subcategory Diagnoses Associated With Fetal Death

Description	Timing of Stillbirth			*P* Value	DeCoDe Level of Certainty^a^		
	Total	Antepartum	Intrapartum		Confident	Probable	Uncertain
Underlying maternal disease or condition attributed to cause of stillbirth	83 (64.3)	61 (63.5)	22 (81.5)	.10	74 (90.2)	5 (6.1)	3 (3.7)
Complications of placenta, cord, and membranes	44 (34.1)	33 (34.4)	11 (40.7)	.65	38 (86.4)	3 (6.8)	3 (6.8)
Other forms of placental separation and hemorrhage	18 (14.0)	10 (10.4)	8 (29.6)		17 (94)	1 (6)	0 (0)
Chorioamnionitis	14 (10.9)	11 (11.5)	3 (11.1)		11 (79)	0 (0)	3 (21)
Other and unspecified morphological and functional abnormalities of placenta	10 (7.8)	10 (10.4)	0 (0)		8 (80)	2 (20)	0 (0)
Other compression of umbilical cord	2 (1.6)	2 (2.1)	0 (0)		2 (100)	0 (0)	0 (0)
Maternal medical and surgical conditions	30 (23.3)	23 (24)	7 (25.9)	.81	29 (96.7)	1 (3.3)	0 (0)
Maternal hypertensive disorders	21 (16.3)	16 (16.7)	5 (18.5)		21 (100)	0 (0)	0 (0)
Other maternal conditions^b^	7 (5.4)	6 (6.2)	1 (3.7)		6 (86)	1 (14)	0 (0)
Maternal infectious and parasitic diseases	1 (0.8)	0 (0)	1 (3.7)		1 (100)	0 (0)	0 (0)
Maternal use of drugs of addiction	1 (0.8)	1 (1)	0 (0)		1 (100)	0 (0)	0 (0)
Other complications of labor and delivery	7 (5.4)	4 (4.2)	3 (11.1)	.18	5 (83.3)	1 (16.7)	0 (0)
Maternal complications of pregnancy	2 (1.6)	1 (1)	1 (3.7)	.39	2 (100)	0 (0)	0 (0)
Premature rupture of membranes	1 (0.8)	0 (0)	1 (3.7)		1 (100)	0 (0)	0 (0)
Other maternal complications of pregnancy	1 (0.8)	1 (1)	0 (0)		1 (100)	0 (0)	0 (0)
Maternal diseases or conditions present but not attributed as an underlying cause of stillbirth^c^	24 (18.6)^d^	21 (21.9)	2 (7.4)	.10	Not evaluated^e^		
Other maternal conditions^f^	11 (8.5)	10 (10.4)	1 (3.7)				
Multiple pregnancy	3 (2.3)	3 (3.1)	0 (0)				
Other and unspecified morphological and functional abnormalities of placenta	3 (2.3)	3 (3.1)	0 (0)				
Maternal hypertensive disorders	2 (1.6)	2 (2.1)	0 (0)				
Maternal infectious and parasitic diseases	1 (0.8)	1 (1)	0 (0)				
Premature rupture of membranes	1 (0.8)	0 (0)	0 (0)				
Malpresentation before labor	1 (0.8)	1 (1)	0 (0)				
Other and unspecified conditions of umbilical cord	1 (0.8)	1 (1)	0 (0)				
Other complications of labor and delivery	1 (0.8)	0 (0)	1 (3.7)				
No maternal condition identified	22 (17.1)^g^	14 (14.6)	3 (11.1)	.76			
Total	129^h^	96	27				

Data are presented as No. (%) unless otherwise indicated.

Abbreviation: DeCoDe, Determination of Cause of Death.

^a^The level of confidence to the diagnosis attributed to by the DeCoDE panel.

^b^One case each of diabetes mellitus in pregnancy, acute vaginitis, primary thrombophilia, infections of genitourinary tract in pregnancy, anemia complicating pregnancy, elderly primigravida and multigravida, pure hypercholesterolemia.

^c^Listing of illnesses considered to be possibly associated with pathogenesis of stillbirth among those cases in which no underlying maternal condition was identified by the DeCoDe panel, and the condition, although present, was not considered to have caused the stillbirth.

^d^Total includes 1 case in which the timing of stillbirth was unknown.

^e^Grading of level of certainty in cause of death attribution not undertaken for possible contributing causes of death.

^f^Human immunodeficiency virus disease complicating pregnancy, childbirth, and the puerperium/antiviral drugs (n = 10); infections of genitourinary tract in pregnancy (n = 1).

^g^Total includes 5 cases in whom the timing of stillbirth was unknown.

^h^Total includes 6 cases in whom the timing of stillbirth was unknown.

Overall, the leading underlying maternal causes of stillbirth were “maternal hypertensive disorders” (16.3%), “other placental separation and hemorrhage” (14.0%; all diagnosed as abruptio placenta), chorioamnionitis (10.9%), and “other morphological and functional abnormality of the placenta” (7.8%; 5 of 6 with placental histology showing evidence of infarcts). Of the 46 cases without an identifiable underlying maternal cause of stillbirth, 34 of the fetuses had an immediate fetal cause of stillbirth attributed (Supplementary Table 3). This included “fetal infection” as the underlying and immediate CoD in 14 (10.9%) cases, all of which were antepartum deaths. Cumulatively, an underlying maternal or immediate fetal condition was diagnosed as the CoD in 90.7% (117/129) of cases.

### Immediate Causes of Fetal Death

The most common immediate fetal causes of stillbirth were fetal infection (37.2%), “antepartum hypoxia/acute intrapartum event” (35.7%), and “congenital malformations/chromosomal abnormalities” (4.7%) (Table 4). The level of certainty in attributing the immediate CoD by the DeCoDe panel (n = 105), was “confident,” “probable,” and “uncertain but possible” in 60.0%, 29.5%, and 9.5%, respectively (Table 4). In 27 (20.9%) cases, the immediate fetal CoD was not determined.

**Table 4.** Immediate Perinatal Cause of Fetal Death

Description	Timing of Stillbirth			*P* Value	DeCoDe Level of Certainty^a^		
	Total	Antepartum	Intrapartum		Confident	Probable	Uncertain
Infection	48 (37.2)	42 (43.8)	6 (22.2)	.047	25 (52.1)	15 (31.2)	8 (16.7)
Sepsis due to *Escherichia coli*	21 (16.3)	20 (20.8)	1 (3.7)	ND^b^	11 (52.4)	6 (28.6)	4 (19.0)
Sepsis due to *Enterococcus* spp	5 (3.9)	5 (5.2)	…	ND^b^	2 (40.0)	3 (60)	…
Congenital CMV infection	5 (3.9)	5 (5.2)	…	ND^b^	5 (100)	…	…
Sepsis due to GBS	4 (3.1)	2 (2.1)	2 (7.4)	ND^b^	3 (75.0)	1 (25.0)	…
Sepsis, unspecified organism	4 (3.1)	2 (2.1)	2 (7.4)	ND^b^	2 (50.0)	1 (25.0)	1 (25.0)
Sepsis due to *Staphylococcus aureus*	2 (1.6)	2 (2.1)	…	ND^b^	1 (50.0)	…	1 (50.0)
Congenital syphilis, unspecified	2 (1.6)	2 (2.1)	…	ND^b^	…	1 (50.0)	1 (50.0)
Other gram-negative sepsis (*Klebsiella pneumoniae*)	1 (0.8)	1 (1.0)	…	ND^b^	…	1 (100)	…
Other specified sepsis^c^	1 (0.8)	1 (1.0)	…	ND^b^	…	…	1 (100)
Other bacterial meningitis^d^	1 (0.8)	1 (1.0)	…	ND^b^	…	1 (100)	…
Congenital pneumonia, unspecified	1 (0.8)	…	1 (3.7)	ND^b^	1 (100)	…	…
Congenital herpes simplex infection	1 (0.8)	1 (1.0)	0 (0)	ND^b^	…	1 (100)	…
Antepartum hypoxia/acute intrapartum event	46 (35.7)	34 (35.4)	12 (44.4)	.50	33 (71.7)	13 (28.3)	…
Congenital malformations, deformations, and chromosomal abnormalities	6 (4.7)	3 (3.1)	3 (11.1)	.12	3 (50.0)	3 (50)	…
Potter syndrome	3 (2.3)	1 (1.0)	2 (7.4)	ND^b^	1 (33.3)	2 (67)	…
Anencephaly	1 (0.8)	0 (0)	1 (3.7)	ND^b^	1 (100)	…	…
Congenital hydrocephalus	1 (0.8)	1 (1.0)	…	ND^b^	1 (100)	…	…
Congenital malformation, unspecified	1 (0.8)	1 (1.0)	…	ND^b^	…	1 (100)	…
Other specified disorder	1 (0.8)	1 (1.0)	…	> .99	1 (100)	…	…
Disorders related to fetal growth	1 (0.8)	1 (1.0)	…	> .99	1 (100)	…	…
Immediate fetal cause of death not determined/unknown	27 (20.9)^e^	15 (15.6)	6 (22.2)	.40	1 (33.3)	…	2 (66.7)
Total	129 (100)^f^	96 (100)	27 (100)		64 (60.0)	31 (29.5)	10 (9.5)

Data are presented as No. (%) unless otherwise indicated.

Abbreviations: CMV, cytomegalovirus; DeCoDe, Determination of Cause of Death; GBS, group B *Streptococcus*; ND, not done.

^a^Level of confidence to the diagnosis attributed to by the DeCoDE panel.

^b^Due to low cell counts, only comparisons for main maternal cause of death were done.

^c^Identified *Ureaplasma urealyticum* and *Escherichia coli* on blood sample.

^d^Culture of *Sphingomonas paucimobilis* on cerebrospinal fluid.

^e^Total includes 6 cases in which the timing of stillbirth was unknown.

^f^Total includes 6 cases in which the timing of stillbirth was unknown.

Thirty-eight of 48 (79.1%) fetal deaths attributed to infection were due to sepsis with the leading implicated organisms being *E. coli* (21/48 [43.8%]), *Enterococcus* species (5/48 [10.4%]), and GBS (4/48 [8.3%]) (Table 4). The overall contribution of these 3 respective pathogens as the cause of stillbirth was 16.3%, 3.9%, and 3.1% (Table 4). The levels of confidence for attributing infection as the cause of fetal death were 52.1%, 31.2%, and 16.7% as “confident,” “probable,” and “uncertain,” respectively (Table 4). Only 2 (1.6%) stillbirths were attributed to syphilis (1 probable and 1 uncertain), whereas 5 (3.9%) were attributed to disseminated congenital CMV infection with high level of confidence, and 1 (0.8%) case to herpes simplex virus (Table 4).

Among the 18 (of 102) stillbirths categorized as SGA, there were no cases where disorders of fetal growth was attributed as the underlying CoD. The specific attributed underlying CoD among these 18 cases included 6 (33.3%) due to “maternal hypertensive conditions” (5 preeclampsia, 1 gestational hypertension without proteinuria); 4 (22.2%) “other and unspecified morphological and functional abnormalities of placenta” (3 placental insufficiency, 1 placental infarction); 1 “other form of placental separation and hemorrhage” (premature separation of the placenta); and 2 “other maternal conditions” (1 unspecified diabetes mellitus in pregnancy and 1 anemia complicating pregnancy). No maternal condition was evident in 5 (27.8%) of the SGA cases, 3 of whom had culture-confirmed fetal infection and 2 of whom had evidence of antepartum hypoxia.

#### Association Between “Underlying” and “Immediate” CoD Attribution

Forty-one of 46 (89.1%) stillbirths with “antepartum hypoxia” as an immediate CoD had an identifiable underlying condition predisposing to hypoxia, foremost among which were placental disorders (n = 24/46 [52.2%]) (Table 5 and Supplementary Table 3). This included 12 cases of “other placental separation and hemorrhage” (26.1%), 8 cases of “other morphological and functional placenta abnormalities” (17.4%), and 2 cases each (4.3%) related to “umbilical cord compression” and “chorioamnionitis.” Furthermore, maternal hypertensive disorders were attributed as the underlying cause of “antepartum hypoxia” in 10 (21.7%) cases.

**Table 5.** Maternal Conditions and Associated Immediate Fetal Cause of Stillbirth

Description^a^	Timing of Stillbirth			DeCODE Level of Certainty^b^		
	Total	Antepartum	Intrapartum	Confident	Probable	Uncertain
Underlying maternal disease or condition affecting fetus	83 (64.3)	61 (63.5)	22 (81.5)	74 (90.2)	5 (6.1)	3 (3.7)
Complications of placenta, cord, and membranes^c^	44 (34.1)	33 (34.4)	11 (40.7)	38 (86.4)	3 (6.8)	3 (6.8)
Fetal antepartum hypoxia/acute intrapartum event	24 (18.6)	18 (18.8)	6 (22.2)	20 (83)	3 (12)	1 (4)
Fetal infection	13 (10.1)	10 (10.4)	3 (11.1)	11 (85)	0 (0)	2 (15)
Disorders related to fetal growth	1 (0.8)	1 (1)	0 (0)	1 (100)	0 (0)	0 (0)
Fetal death of unspecified cause	6 (4.7)	4 (4.2)	2 (7.4)	6 (100)	0 (0)	0 (0)
Maternal medical and surgical conditions^c^	30 (23.3)	23 (24)	7 (25.9)	29 (96.7)	1 (3.3)	0 (0)
Fetal antepartum hypoxia/acute intrapartum event	15 (11.6)	11 (11.5)	4 (14.8)	14 (93)	1 (7)	0 (0)
Fetal infection	9 (7)	8 (8.3)	1 (3.7)	9 (100)	0 (0)	0 (0)
Other specified fetal disorder	1 (0.8)	1 (1)	0 (0)	1 (100)	0 (0)	0 (0)
Fetal death of unspecified cause	5 (3.9)	3 (3.1)	2 (7.4)	5 (100)	0 (0)	0 (0)
Other complications of labor and delivery^c^	7 (5.4)	4 (4.2)	3 (11.1)	5 (83.3)	1 (16.7)	0 (0)
Fetal antepartum hypoxia/acute intrapartum event	1 (0.8)	0 (0)	1 (3.7)	1 (100)	0 (0)	0 (0)
Fetal congenital malformations, deformations, and chromosomal abnormalities	1 (0.8)	1 (1)	0 (0)	1 (100)	0 (0)	0 (0)
Fetal infection	1 (0.8)	1 (1)	0 (0)	1 (100)	0 (0)	0 (0)
Fetal death of unspecified cause	4 (3.1)	2 (2.1)	2 (7.4)	2 (67)	1 (33)	0 (0)
Maternal complications of pregnancy^c^	2 (1.6)	1 (1)	1 (3.7)	2 (100)	0 (0)	0 (0)
Fetal antepartum hypoxia/acute intrapartum event	1 (0.8)	1 (1)	0 (0)	1 (100)	0 (0)	0 (0)
Fetal infection	1 (0.8)	0 (0)	1 (3.7)	1 (100)	0 (0)	0 (0)
Coincidental maternal diseases or conditions associated with stillbirths^c^	24 (18.6)	21 (21.9)	2 (7.4)	Not evaluated^b^		
Fetal infection	14 (10.9)	13 (13.5)	1 (3.7)			
Fetal antepartum hypoxia/acute intrapartum event	3 (2.3)	3 (3.1)	0 (0)			
Fetal congenital malformations, deformations, and chromosomal abnormalities	1 (0.8)	0 (0)	1 (3.7)			
Fetal death of unspecified cause	6 (4.7)	5 (5.2)	0 (0)			
No maternal condition^c,d^	22 (17.1)	14 (14.6)	3 (11.1)			
Fetal infection	10 (7.8)	10 (10.4)	0 (0)			
Fetal congenital malformations, deformations, and chromosomal abnormalities	4 (3.1)	2 (2.1)	2 (7.4)			
Fetal antepartum hypoxia/acute intrapartum event	2 (1.6)	1 (1)	1 (3.7)			
Fetal death of unspecified cause	6 (4.7)	1 (1)	0 (0)			
Total	129	96	27			

Abbreviation: DeCoDe, Determination of Cause of Death.

^a^Listing of illnesses considered to be possibly associated with pathogenesis of stillbirth among those cases in which no underlying maternal condition was identified by the DeCoDe panel and the condition, although present, was not considered to have caused the stillbirth.

^b^Grading of level of certainty in cause of death attribution not undertaken for possible contributing causes of death.

^c^Main row headings are underlying maternal conditions with immediate fetal conditions given below.

^d^Absence of an underlying maternal condition associated with stillbirths.

Among the 48 stillbirths attributed to fetal infection (including sepsis, pneumonia, or meningitis) as the immediate cause, sepsis was the underlying CoD in 10 (20.8%) cases among whom no maternal condition was identified, whereas “chorioamnionitis” and “maternal hypertensive disorders” were the underlying diagnoses in 12 (25.0%) and 9 (18.8%) cases with fetal infection, respectively. Four of 6 (66.7%) stillbirths with congenital abnormalities as an immediate CoD were assessed to have died due to the congenital abnormality in the absence of any underlying maternal condition (Supplementary Table 3). Although no immediate CoD was attributable to 27 stillbirths, an underlying condition was present in 15, of which 4 (14.8%) were due to “maternal hypertensive disorders” and 6 (22.2%) due to “complications of placenta, cord, and membranes.”

## DISCUSSION

This study highlights the potential of blood, lung, and CSF microbial culture and molecular tests (including on organ tissue samples), coupled with maternal clinical medical record review, and macroscopic and histological placenta examination, in ascertaining the causes of stillbirth in an LMIC setting. A cause of stillbirth was identifiable for 90.7% of the 129 stillbirths investigated, including an underlying maternal cause in 63.4% and an immediate fetal cause in 79.1% of cases. The main objective of this pilot study that preceded the multicenter Child Health and Mortality Prevention Surveillance (CHAMPS) program currently under way was to evaluate the utility of MITS as a diagnostic tool, rather than provide conclusive data on the causes of stillbirths. Nevertheless, in addition to indicating the potential of our approach in ascertaining granular causes of stillbirth in LMIC settings, the findings provide insight into the pathogenesis of stillbirths in settings such as ours. This included the potentially underappreciated dominant role of fetal infection as the cause of stillbirth in 37.2% of cases. The leading bacteria implicated in these deaths were *E. coli* (16.3%), *Enterococcus* species (3.9%), and GBS (3.1%). In the majority of stillbirths due to sepsis, pneumonia, or meningitis (n = 40), an underlying maternal condition that likely compromised placental integrity and predisposed to such infection was identified. This included “histologically confirmed chorioamnionitis” (n = 9 [22.5%]) and “maternal hypertensive or other disorders” (n = 6 [15%]).

Although fetal bacterial infection is a recognized cause of stillbirth [1, 12, 13], its relative role and characterization of the specific organisms has largely been ill defined in LMICs due to the absence of systematic investigation of these deaths [2]. Illustrative of this is a modeling simulation of stillbirth causes in LMICs, which estimated that 32% of deaths were caused by infections in sub-Saharan Africa. These infections, however, were attributed to malaria (20%), syphilis (11%), and HIV infection (1%), with no mention of the fetal invasive bacterial infection [1]. In our study, which was done in a malaria-free and high maternal HIV prevalence (29%) setting, only 1.6% of stillbirths were attributed to syphilis (despite 5.8% of women screening positive during pregnancy), and none of the cases were attributed to maternal HIV infection.

The recent Mozambican study, which validated MIA to CDA, attributed 22.2% (n = 4) of 18 stillbirths to fetal sepsis; 3 of which were due to GBS [7]. The absence of considering maternal medical records or placenta evaluation in the Mozambican study, however, precluded an assessment on whether underlying maternal or placental conditions predisposed to these infections as is evident in our study. Furthermore, intrauterine growth restriction was attributed as the cause of stillbirth in 39% of cases in the Mozambican study, without any explanation for the possible underlying cause thereof. In contrast, although 17.6% of stillbirths in our study were assessed as SGA, only 1 death was attributed to a disorder of fetal growth as the immediate CoD (in which “placental morphological and functional abnormality” was identified as the underlying cause). An identifiable, underlying maternal or placental condition was attributed as the cause of stillbirth in 72.2% of stillbirths with SGA. These findings further emphasize the importance of maternal medical history and need for placenta investigation for an in-depth understanding on the causes of stillbirth in LMICs. A more granular understanding of the pathway to fetal death would be instrumental in determining which underlying factors might be modifiable during pregnancy and labor, in the absence of which strategies aimed at preventing these deaths might be misdirected.

Although this was a hospital, facility-based study in a relatively well-resourced setting compared to most other sub-Saharan African countries, antepartum hypoxia (35.7%) was still the leading immediate cause of stillbirths. Our systematic investigation of the cases, however, enabled us to identify the underlying maternal conditions that led to the asphyxia in the majority of the cases. This included intrauterine asphyxia secondary to placenta abnormalities and maternal medical conditions such as hypertension. Such data assist in prioritizing interventions that might prevent these deaths.

Our study observed a low yield of information from histopathology investigation of core biopsies of various solid tissue organs targeted for investigation; this despite MITS being done at a median of 16.8 hours postdelivery, albeit presentation to the hospital was 9.1 hours beforehand and the exact time since death was unknown for antepartum cases. The high number of autolyzed samples (41%–65%) could possibly be due to the lifeless fetus having been in utero at 37°C for a lengthy period. Furthermore, the MITS was undertaken using a standardized approach and without any sonar guidance, which could have contributed to the high percentage of cases in which tissue sampling was either inadequate or no target tissue sample was obtained at all. As such, these data indicate the limitation of core tissue sampling for histopathology examination, especially of macerated stillbirths. The yield of adequate tissue sampling among the stillbirth cases has not been reported on in the Mozambican study [7]. The “blind” MITS approach used, however, provided adequate tissue for histopathology examination among the majority of the neonatal deaths that were investigated as part of the broader study (76.8% with adequate samples, 8.4% with suboptimal number of core samples) and among whom only 0.6% of samples were autolyzed as reported elsewhere in this supplement [8].

Other limitations of our study include retrospective review of maternal medical records, and failure to retrieve the placenta for macroscopic and histopathology examination in 23.3% of cases, which could have undermined our attribution on the underlying CoD for some cases. In addition, we opportunistically used commercially available molecular assays designed for investigation of viral meningoencephalitis and neonatal sepsis, rather than assays designed specifically to investigate for organisms associated with stillbirths, although there is some overlap.

Another challenge of this study was the interpretation of the repertoire of data available for CoD ascertainment. We chose to determine the causes of stillbirth through deliberation of individual cases by an expert panel. Although this exercise was more intensive than, as an example, that employed in SCRN study in which cases were deliberated upon by 2 obstetricians [6], it does raise issues on how best to interpret and analyze data to enable comparisons between studies. Recent epidemiological studies in LMICs have developed computer-based hierarchal algorithms for assigning cause of stillbirth. The data underpinning these algorithms were, however, limited to maternal medical record review. The algorithm attributed a cause to 82.9% of the stillbirths, with fetal asphyxia (46.6%) associated with prolonged or obstructed labor (19%), preeclampsia (18%), and antepartum hemorrhage (18%) as the leading cause of death. In addition, infection was attributed as the cause of death in 20.8% of the cases. The absence of biological investigations of these cases, however, prevented ascertainment as to the specific causes of these infections, as well as defining whether underlying placenta abnormalities contributed to the pathogenesis of these infections [14]. Whether such algorithms can be developed with additional layered information such as placental histopathology and culture and molecular test results from stillbirths warrants further consideration. The recently launched CHAMPS project is a case example through which this could be achieved [15].

In conclusion, this study highlights the potential of maternal medical record review, coupled with targeted MITS and placental macroscopic and histological examination in providing in-depth insight into the underlying and immediate causes of stillbirths in a setting where the stillbirth rate was 23 per 1000 births. Similar data are required from a diversity of settings to better characterize the research agenda and interventions that are required, to achieve the global target of reducing stillbirth rates to 12 per 1000 by 2030 [4].

## Supplementary Data

Supplementary materials are available at *Clinical Infectious Diseases* online. Consisting of data provided by the authors to benefit the reader, the posted materials are not copyedited and are the sole responsibility of the authors, so questions or comments should be addressed to the corresponding author.

ciz573_suppl_Supplementary_Material

## Notes

***Author contributions.*** Conceptualization and methodology, supervision, funding acquisition, and writing of the first draft: S. A. M. Data curation: S. A. M., R. C., J. P., V. B., A. I., S. N., F. S., A. M., M. H., S. Z., J. W., Y. A., C. C. Data analysis: S. A. M., R. C., A. I., S. J. Laboratory testing: V. B., M. H., S. Z., J. W. Project administration: A. M., S. N., R. C. Training of staff on minimally invasive tissue sampling: Q. B., J. O. Revisions and editing: S. A. M., J. P., V. B., C. C., Y. A., A. I., Q. B., D. M. B., R. F. B., M. H., S. J., R. M., A. M., S. N., J. O., P. L. R., J. M. R., F. S., J. W., S. Z., R. C.

***Acknowledgments.*** The authors acknowledge the important contributions of the Determining Cause of Death (DeCoDe) panel (speciality in parenthesis) that was chaired by Scott Dowell (epidemiologist, pediatrician) and Christopher Wilson (infectious diseases, pediatrician). Other DeCoDe panel members were as follows: Quique Bassat (infectious diseases, pediatrician), Dianna Blau (microbiology), Robert Breiman (epidemiology, infectious diseases, physician), Daniel Chandramohan (epidemiology, World Health Organization *International Classification of Diseases* coding specialist), Richard Chawana (physician), Kevin Clarke (microbiology), Martin Hale (pathology), Penny Heaton (physician), Eric Houpt (microbiology, infectious disease, physician), Aggrey Igunza (study clinician), Grace Irimu (pediatrician), Reinhard Kaiser (epidemiology, physician), Nicholas Kassebum (microbiology), Keith Klugman (microbiology, infectious diseases, physician), Karen Kotloff (infectious diseases, pediatrician), Shabir A. Madhi (infectious diseases, pediatrician), Kim Mulholland (infectious diseases, epidemiology, pediatrician), Jaume Ordi (pathology), Robert Pattinson (obstetrician), Rebecca Philipsborn (pediatrician), Pratima Raghunathan (epidemiology), Anna Seale (infectious diseases, pediatrician), Milagritos Tapia (infectious diseases, pediatrician), Allan Taylor (epidemiology, pediatrician), Sherif Zaki (pathology), and Reena Mohanlal, Sugeshnee Pather, Marie Suleman, Eunice Van Den Berg, and Reubina Wadee (Anatomical Pathology, National Health Laboratory Service of South Africa).

***Financial support.*** This work was supported by the Bill & Melinda Gates Foundation [OPP1101764].

***Supplement sponsorship.*** This supplement is sponsored by the Emory Global Health Institute and the Bill & Melinda Gates Foundation.

***Potential conflicts of interest.*** S. A. M. has received grants from the Bill & Melinda Gates Foundation, Pfizer, Glaxo Smith Kline, Biovac, Novavax, and Sanofi Pasteur; and honoraria from The Bill & Melinda Gates Foundation. All other authors report no potential conflicts of interest. All authors have submitted the ICMJE Form for Disclosure of Potential Conflicts of Interest. Conflicts that the editors consider relevant to the content of the manuscript have been disclosed.
