# Supplementary material for: An Observational Pilot Study Evaluating the Utility of Minimally Invasive Tissue Sampling to Determine the Cause of Stillbirths in South African Women
Source: Clin Infect Dis. 2019 Oct 9;69(Suppl 4):S342–50. doi: 10.1093/cid/ciz573 (PMC6785671; doi:10.1093/cid/ciz573)
Supplement: ciz573_suppl_Supplementary_Material [file ciz573_suppl_supplementary_material.docx]

**Supplementary material for Stillbirths**

***Cause of death attribution***

The Determining Cause of Death (DeCoDe) panel convened from 26^th^ March to 5^th^ April 2017 in South Africa. The panel composed pathologists, paediatricians, epidemiologists, microbiologists, infectious disease specialists and an obstetrician. The results from the entire database, including individual patient records were made available for review. Following a summarised presentation of each case by either SAM or RC, the panel under the Chair of either Chris Wilson or Scott Dowell, deliberated on the cause of stillbirth. The framework for reporting on the cause of stillbirth was based on the 2016/7 WHO perinatal CoD reporting recommendations.[1] This included recording the “underlying condition”, i.e. the most distal condition that initiated the sequence of events leading to the stillbirth; with precedence given to identifiable maternal over foetal conditions. Furthermore, the most proximal event (i.e. immediate CoD) leading to foetal death, if different from the underlying cause was also recorded; as were any other condition noted in the causal pathway chain to foetal death. To reflect the DeCoDE panel’s confidence in attributing the CoD, a certainty score between level- 1 (confident), level- 2 (probable) and level- 3 (uncertain) was assigned for the “immediate” and “underlying” diagnoses. Case examples of how the CoD was attributed among stillbirths are illustrated below:

An example of cause of stillbirth attribution included a fetus that developed intra-uterine fetal growth retardation, as a consequence of placental infarction possibly due to the pre-eclampsia, with evidence of intrauterine asphyxia. The underlying cause of death (CoD) would be attributed to pre-eclampsia, with placental infarction as an antecedent condition, whilst intra-uterine asphyxia would be listed as the “immediate” CoD. In this instance, the fetal growth retardation could be listed either as an additional antecedent condition, consequent to the placental infarction, or could be listed as a contributing co-morbidity because it was considered not to have been directly related to the death.

Another example would be a mother with no identifiable predisposing cause for a stillbirth, who delivers a stillbirth in whom Group B streptococcus (GBS) is identified from a usually sterile site, e.g. blood culture. In this instance, the invasive GBS disease would be recorded as the underlying and immediate CoD.

The CoD were coded by a medical doctor (Fatima Solomon) using the 10th Revision of International Classification of Diseases and Related Health Problems and Perinatal Deaths (ICD-10 and ICD-10 PM) coding systems.

**Supplementary table 1: Placenta macroscopic and microscopic investigation from stillbirths**

| **Characteristic** | **Overall**  **N=99** | **Antepartum N=77** | **Intrapartum**  **N=21** | **P value** |
| --- | --- | --- | --- | --- |
| Median Placenta weight– grams (range) | 365 (121-820) | 359 (121-655) | 383 (161-820) | 0.22 |
| Placenta birthweight ratio (SD) ^a^ | 6.00 (2.29) | 5.92 (1.98) | 5.79 (2.12) | 0.81 |
| **Umbilical cord (%)** |  |  |  |  |
| Cord insertion: central | 41/93 (44.1) | 33/76 (43.4) | 7/16 (43.8) |  |
| Eccentric | 50/93 (53.8) | 41/76 (53.9) | 9/16 (56.2) |  |
| Marginal | 2/93 (2.2) | 2/76 (2.6) | 0/16 (0) | >0.99 |
| Number of cord vessels: 3 | 96/96 (100) | 74/74 (100) | 21/21 (100) | >0.99 |
| Cord thrombosis | 4/95 (4.2) | 3/75 (4) | 1/20 (5) | >0.99 |
| Cord knots | 4/95 (4.2) | 3/75 (4) | 0/19 (0) | >0.99 |
| Funistis | 3/97 (3.1) | 2/75 (2.7) | 1/21 (4.8) | 0.53 |
| Chorionic vasculitis | 2/91 (2.2) | 2/72 (2.8) | 0/19 (0) | >0.99 |
| **Placenta membranes (%)** |  |  |  |  |
| Membrane Colour: Translucent | 79/95 (83.2) | 59/73 (80.8) | 19/21 (90.5) |  |
| Other | 16/95 (16.8) | 14/73 (19.2) | 2/21 (9.5) | 0.51 |
| Amnion nodusum | 1/92 (1.1) | 1/73 (1.4) | 0/19 (0) | >0.99 |
| **Chorioamnionitis (%)** | 27/98 (27.6) | 22/76 (28.9) | 4/21 (19) | 0.42 |
| Grading | N =22 | N=19 | N=3 |  |
| Grade I (<10 cells/phf) | 7/22 (31.8) | 6/19 (31.6) | 1/3 (33.3) |  |
| Grade II (10-20 cells/phf) | 9/22 (40.9) | 9/19 (47.4) | 0/3 (0) |  |
| Grade III (>30 cells/phf) | 6/22 (27.3) | 4/19 (21.1) | 2/3 (66.7) | 0.16 |
| Acute chorioamnionitis | 18/18 (100) | 15/15 (100) | 3/3 (100) | >0.99 |
| **Placenta parenchyma (%)** |  |  |  |  |
| Macroscopic infarct | 26/99 (26.3) | 19/77 (24.7) | 7/21 (33.3) | 0.42 |
| Colour: Normal | 29/99 (29.3) | 21/77 (27.3) | 7/21 (33.3) |  |
| Pale | 26/99 (26.3) | 21/77 (27.3) | 5/21 (23.8) |  |
| Congested | 44/99 (44.4) | 35/77 (45.5) | 9/21 (42.9) | 0.86 |
| Retroplacental hematoma (%) | 30/96 (31.2) | 23/75 (30.7) | 6/20 (30) | >0.99 |
| **Placenta cut surface findings** |  |  |  |  |
| Infarction (%) | 46/92 (50) | 37/73 (50.7) | 9/19 (47.4) | >0.99 |
| <10% | 5/38 (13.2) | 3/30 (10) | 2/8 (25) |  |
| 10-<20% | 22/38 (57.9) | 19/30 (63.3) | 3/8 (37.5) |  |
| 20-<30% | 7/38 (18.4) | 5/30 (16.7) | 2/8 (25) |  |
| >=30% | 4/38 (10.5) | 3/30 (10) | 1/8 (12.5) | 0.46 |
| Infarct old | 19/20 (95) | 16/17 (94.1) | 3/3 (100) |  |
| Infarct new | 1/20 (5) | 1/17 (5.9) | 0/3 (0) | >0.99 |
| Intervillous fibrin | 11/94 (11.7) | 8/74 (10.8) | 2/19 (10.5) | >0.99 |
| Other chorionic plate abnormalities | 3/99 (3) | 3/77 (3.9) | 0/21 (0) | >0.99 |
| Other decidua basalis abnormalities | 3/99 (3) | 1/77 (1.3) | 2/21 (9.5) | 0.12 |

^a^Calculated for those with birthweight and placenta weight (N=97).

**Supplementary table 2: Tissue sample adequacy for histology**

| Sample | Adequate  N (%) | Autolysed  N (%) | No sample submitted  N (%) | Suboptimal sample  N (%) | Total |
| --- | --- | --- | --- | --- | --- |
| Left lung | 2 (1.6) | 71 (55.5) | 53 (41.4) | 2 (1.6) | 128 (100) |
| Right lung | 2 (1.6) | 83 (64.8) | 42 (32.8) | 1 (0.8) | 128 (100) |
| Liver | 2 (1.6) | 53 (41.4) | 17 (13.3) | 56 (43.8) | 128 (100) |
| Brain | 3 (2.3) | 52 (40.6) | 71 (55.5) | 2 (1.6) | 128 (100) |

**Supplementary table 3 Association between the maternal condition and most immediate identified cause of all stillbirths.**

| **Maternal conditions^a^** | **Immediate fetal cause of death** | | | | | | | | | | | | | | | | | | | | | | |
| --- | --- | --- | --- | --- | --- | --- | --- | --- | --- | --- | --- | --- | --- | --- | --- | --- | --- | --- | --- | --- | --- | --- | --- |
|  | **Infection** | Sepsis due to Escherichia coli | Sepsis due to Enterococcus | Congenital cytomegalovirus infection | Sepsis due to Group B streptococcus | Sepsis, unspecified organism | Sepsis due to Staphylococcus aureus | Congenital syphilis, unspecified | Other gram negative sepsis (Klebsiella pneumoniae) | Other specified sepsis | Other bacterial meningitis | Congenital pneumonia, unspecified | Congenital herpes simplex infection | **Antepartum hypoxia/Acute intrapartum event** | **Congenital malformations, deformations and chromosomal abnormalities** | Potter’s syndrome | Anencephaly | Congenital hydrocephalus | Congenital malformation, unspecified | **Other specified disorder** | **Disorders related to fetal growth** | **Death of unspecified cause** | **Total** |
| ***Underlying maternal condition*** | 24 | 10 | 1 | 2 | 3 | 3 | 1 | 1 | 0 | 0 | 1 | 1 | 1 | 41 | 1 | 0 | 0 | 1 | 0 | 1 | 1 | 15 | **83 (64.3)** |
| **Complications of placenta, cord and membranes** | 13 | 4 | 0 | 1 | 3 | 2 | 0 | 1 | 0 | 0 | 1 | 0 | 1 | 24 | 0 | 0 | 0 | 0 | 0 | 0 | 1 | 6 | **44 (34.1)** |
| Other forms of placental separation and hemorrhange | 1 | 1 | 0 | 0 | 0 | 0 | 0 | 0 | 0 | 0 | 0 | 0 | 0 | 12 | 0 | 0 | 0 | 0 | 0 | 0 | 0 | 5 | **18 (14.0)** |
| Chorioamnionitis | 12 | 3 | 0 | 1 | 3 | 2 | 0 | 1 | 0 | 0 | 1 | 0 | 1 | 2 | 0 | 0 | 0 | 0 | 0 | 0 | 0 | 0 | **14 (10.9)** |
| Other and unspecified morphological and functional abnormalities of placenta | 0 | 0 | 0 | 0 | 0 | 0 | 0 | 0 | 0 | 0 | 0 | 0 | 0 | 8 | 0 | 0 | 0 | 0 | 0 | 0 | 1 | 1 | **10 (7.8)** |
| Other compression of umbilical cord | 0 | 0 | 0 | 0 | 0 | 0 | 0 | 0 | 0 | 0 | 0 | 0 | 0 | 2 | 0 | 0 | 0 | 0 | 0 | 0 | 0 | 0 | **2 (1.6)** |
| **Maternal medical and surgical conditions** | 9 | 5 | 1 | 1 | 0 | 0 | 1 | 0 | 0 | 0 | 0 | 1 | 0 | 15 | 0 | 0 | 0 | 0 | 0 | 1 | 0 | 5 | **30 (23.3)** |
| Maternal hypertensive disorders | 7 | 5 | 0 | 1 | 0 | 0 | 0 | 0 | 0 | 0 | 0 | 1 | 0 | 10 | 0 | 0 | 0 | 0 | 0 | 0 | 0 | 4 | **21 (16.3)** |
| Other maternal conditions | 2 | 0 | 1 | 0 | 0 | 0 | 1 | 0 | 0 | 0 | 0 | 0 | 0 | 4 | 0 | 0 | 0 | 0 | 0 | 1 | 0 | 0 | **7 (5.4)** |
| Mmaternal infectious and parasitic diseases | 0 | 0 | 0 | 0 | 0 | 0 | 0 | 0 | 0 | 0 | 0 | 0 | 0 | 1 | 0 | 0 | 0 | 0 | 0 | 0 | 0 | 0 | **1**  **(0.8)** |
| Maternal use of drugs of addiciton | 0 | 0 | 0 | 0 | 0 | 0 | 0 | 0 | 0 | 0 | 0 | 0 | 0 | 0 | 0 | 0 | 0 | 0 | 0 | 0 | 0 | 1 | **1**  **(0.8)** |
| **Other complications of labour and delivery** | 1 | 1 | 0 | 0 | 0 | 0 | 0 | 0 | 0 | 0 | 0 | 0 | 0 | 1 | 1 | 0 | 0 | 1 | 0 | 0 | 0 | 4 | **7**  **(5.4)** |
| Other complications of labour and delivery | 1 | 1 | 0 | 0 | 0 | 0 | 0 | 0 | 0 | 0 | 0 | 0 | 0 | 1 | 1 | 0 | 0 | 1 | 0 | 0 | 0 | 4 | **7**  **(5.4)** |
| **Maternal complications of pregnancy** | 1 | 0 | 0 | 0 | 0 | 1 | 0 | 0 | 0 | 0 | 0 | 0 | 0 | 1 | 0 | 0 | 0 | 0 | 0 | 0 | 0 | 0 | **2**  **(1.6)** |
| Premature rupture of membranes | 1 | 0 | 0 | 0 | 0 | 1 | 0 | 0 | 0 | 0 | 0 | 0 | 0 | 0 | 0 | 0 | 0 | 0 | 0 | 0 | 0 | 0 | **1**  **(0.8)** |
| Other maternal complications of pregnancy | 0 | 0 | 0 | 0 | 0 | 0 | 0 | 0 | 0 | 0 | 0 | 0 | 0 | 1 | 0 | 0 | 0 | 0 | 0 | 0 | 0 | 0 | **1**  **(0.8)** |
| ***Coincidental conditions^b^*** | 14 | 6 | 2 | 3 | 1 | 1 | 0 | 1 | 0 | 0 | 0 | 0 | 0 | 3 | 1 | 1 | 0 | 0 | 0 | 0 | 0 | 6 | **24 (18.6)** |
| **Other maternal conditions** | 5 | 2 | 0 | 2 | 0 | 1 | 0 | 0 | 0 | 0 | 0 | 0 | 0 | 2 | 1 | 1 | 0 | 0 | 0 | 0 | 0 | 3 | **11 (8.5)** |
| Multiple pregnancy | 1 | 0 | 0 | 0 | 0 | 0 | 0 | 1 | 0 | 0 | 0 | 0 | 0 | 0 | 0 | 0 | 0 | 0 | 0 | 0 | 0 | 2 | **3**  **(2.3)** |
| Other and unspecified morphological and functional abnormalities of placenta | 2 | 1 | 1 | 0 | 0 | 0 | 0 | 0 | 0 | 0 | 0 | 0 | 0 | 1 | 0 | 0 | 0 | 0 | 0 | 0 | 0 | 0 | **3**  **(2.3)** |
| Maternal hypertensive disorders | 2 | 1 | 0 | 1 | 0 | 0 | 0 | 0 | 0 | 0 | 0 | 0 | 0 | 0 | 0 | 0 | 0 | 0 | 0 | 0 | 0 | 0 | **2**  **(1.6)** |
| Maternal infectious and parasitic diseases | 1 | 0 | 1 | 0 | 0 | 0 | 0 | 0 | 0 | 0 | 0 | 0 | 0 | 0 | 0 | 0 | 0 | 0 | 0 | 0 | 0 | 0 | **1**  **(0.8)** |
| Premature rupture of membranes | 0 | 0 | 0 | 0 | 0 | 0 | 0 | 0 | 0 | 0 | 0 | 0 | 0 | 0 | 0 | 0 | 0 | 0 | 0 | 0 | 0 | 1 | **1**  **(0.8)** |
| Malpresentation before labour | 1 | 1 | 0 | 0 | 0 | 0 | 0 | 0 | 0 | 0 | 0 | 0 | 0 | 0 | 0 | 0 | 0 | 0 | 0 | 0 | 0 | 0 | **1**  **(0.8)** |
| Other and unspecified conditions of umbilical cord | 1 | 1 | 0 | 0 | 0 | 0 | 0 | 0 | 0 | 0 | 0 | 0 | 0 | 0 | 0 | 0 | 0 | 0 | 0 | 0 | 0 | 0 | **1**  **(0.8)** |
| Other complications of labour and delivery | 1 | 0 | 0 | 0 | 1 | 0 | 0 | 0 | 0 | 0 | 0 | 0 | 0 | 0 | 0 | 0 | 0 | 0 | 0 | 0 | 0 | 0 | **1**  **(0.8)** |
| ***No maternal condition^c^*** | 10 | 5 | 2 | 0 | 0 | 0 | 1 | 0 | 1 | 1 | 0 | 0 | 0 | 2 | 4 | 2 | 1 | 0 | 1 | 0 | 0 | 6 | **22 (17.1)** |
| **Total** | **48 (37.2)** | **21 (16.3)** | **5 (3.9)** | **5 (3.9)** | **4 (3.1)** | **4 (3.1)** | **2 (1.6)** | **2 (1.6)** | **1 (0.8)** | **1 (0.8)** | **1 (0.8)** | **1 (0.8)** | **1 (0.8)** | **46 (35.7)** | **6 (4.7)** | **3 (2.3)** | **1 (0.8)** | **1 (0.8)** | **1 (0.8)** | **1 (0.8)** | **1 (0.8)** | **27 (20.9)** | **129 (100.0)** |

^a^ Conditions indicated in bold are high level conditions with specific conditions given below. ^b^ Listing of illnesses considered to be possibly associated with pathogenesis of stillbirth among those cases in which no underlying maternal condition was identified by the Determination of Cause of Death (DeCoDe) panel; and the condition although present was not considered to have caused the stillbirth. ^c^Absence of an underlying maternal condition associated with stillbirths.

References

1. World Health Organization. The WHO application of ICD-10 to deaths during the perinatal period. 2016: 1–88. Available at: http://www.who.int/reproductivehealth/publications/monitoring/icd-10-perinatal-deaths/en/. Accessed 29 August 2018.
